# Patient Perspectives Toward a Decision Aid for Radioactive Iodine Treatment for Intermediate Risk Thyroid Cancer

**DOI:** 10.1007/s12529-025-10408-4

**Published:** 2025-12-18

**Authors:** Alaina L. Carr, Kate L. Gabriel, Gautham Pillai, James Pocchia, Kristi D. Graves, Jacqueline Jonklaas

**Affiliations:** 1 Georgetown University Medical Center, Department of Oncology, Washington, DC, United States; 2 Georgetown University School of Health, Department of Health Systems Administration, Washington, DC, United States; 3 Georgetown University School of Health, Department of Human Science, Washington, DC, United States; 4 Division of Endocrinology, Georgetown University, Washington, DC, United States

**Keywords:** Cancer, Decision support techniques, Iodine radioisotopes, Oncology, Patient-centered care, Thyroid neoplasm

## Abstract

**Background:**

Patients with intermediate risk thyroid cancer face the decision of whether or not to undergo radioactive iodine (RAI) treatment after total thyroidectomy. This process is challenging due to the unclear risks and benefits of RAI treatment for intermediate risk disease. The study identified the decisional needs of patients offered RAI treatment for thyroid cancer to inform the development of a web-based patient decision aid (PtDA).

**Method:**

We used purposive sampling to recruit 23 adult patients with thyroid cancer (*M*_age_ = 39.1; 83% female) from three metropolitan hospitals who were offered RAI treatment. Participants completed an online survey before taking part in one of four 2-h focus groups moderated by a clinical psychologist. Semi-structured interviews explored patients’ experiences, decisional needs, and recommended elements for a PtDA. Two raters independently coded transcripts and used content analysis to analyze qualitative data.

**Results:**

Content analysis revealed three broad domains: (1) the range of patient involvement in the RAI treatment decision-making process, (2) personal values-based decisional outcomes, and (3) decision aid content recommendations on the basis of patients’ knowledge gaps about RAI treatment. Patients’ recommendations included the need for information on the RAI dose and common side effects, risk stratification, safety precautions for radioactivity, low-iodine diet guidance, and financial costs.

**Conclusion:**

The study provides patient insights for a targeted web-based PtDA that integrates personal values, risk information, and logistical considerations to support informed decision-making about RAI treatment. Future research to examine the benefits of PtDAs for treatment of intermediate-risk thyroid cancer is needed.

## Introduction

Thyroid cancer has excellent 5-year survival outcomes due to early detection and effective treatments [1–4]. First-line treatment includes thyroidectomy surgery or removal of the thyroid gland, followed by radioactive iodine (RAI) treatment or careful monitoring without RAI [2–7]. RAI treatment recommendations depend on disease severity and the risk of recurrence. RAI is not recommended for patients with low-risk thyroid cancer and is strongly recommended for patients with high-risk thyroid cancer, as high-risk patients have a reduced risk of disease recurrence after RAI treatment [8, 9]. However, treatment benefits are uncertain for patients with intermediate risk thyroid cancer.

Among the 860,000 thyroid cancer survivors in the USA in 2020, approximately 35% had intermediate risk disease [10, 11]. The management of patients with intermediate disease presents a challenge, as there is a wide spectrum of disease severities and no definitive guidelines on whether RAI should be administered. Data from 2023 indicates that approximately 69% of patients with intermediate risk thyroid cancer receive RAI treatment [12]. More recently, in the 2025 American ATA clinical practice guidelines, intermediate risk is now categorized into two subcategories (intermediate low and intermediate high risk), and for both of these risk categories, the ATA recommends the *consideration* of RAI treatment for intermediate risk patients, with an emphasis on patient preference due to low certainty evidence [2, 8, 9, 13, 14]. This lack of consensus makes treatment decisions highly individualized. Factors such as patient age, fertility considerations, comorbidities, and differences across physicians and institutional practices contribute to the RAI treatment decision-making process among intermediate risk patients. Thus, the decision process regarding RAI treatment is more complex for intermediate low and intermediate high risk thyroid cancer (hereafter “intermediate risk”) than for other thyroid cancer risk types.

When no single treatment option is considered the “optimal” choice, shared decision-making, where patients and their providers work together to understand the benefits and risks of different treatment options on the basis of patient preferences, becomes imperative [15, 16]. However, the evidence to date suggests that shared decision-making about RAI treatment may not always occur. For example, in a large population-based study, 55.8% of patients reported feeling that they were not provided a choice about whether to undergo RAI treatment [5]. A lack of perceived choice regarding RAI was associated with a higher rate of RAI treatment and lower treatment satisfaction [5]. In contrast, patients actively involved in the shared decision-making process and receiving clear information about the risks and benefits of RAI reported less decisional regret about their treatment choice [17–19]. Unfortunately, tools to support shared decision-making in intermediate risk thyroid cancer patients remain scarce despite the growing emphasis on person-centered communication in clinical guidelines [8, 18] and healthcare organizations [20, 21].

Decision support tools such as patient decision aids (PtDAs) may address this gap by providing patients with information on treatment options and the risks and benefits of each option [8, 21, 22]. PtDAs are tools that provide factual information about the condition and define the treatment decision to be made, clarify risk management options, explain risks and benefits of each option, help patients clarify their own attitudes about the treatment options, and provide guidance on how to use the information (e.g., bringing the information into a discussion with one’s medical team) to reach a decision [23]. Robust evidence indicates that PtDAs improve patients’ medical knowledge, reduce decisional conflict, improve decision quality, and lead to more informed treatment choices across various cancers [16, 24–29]. In research conducted before 2016 when patients with low-risk thyroid cancer still received RAI treatment [30], a small randomized controlled trial in Canada evaluated a PtDA and found the PtDA led to increased knowledge and decreased decisional conflict in low-risk patients considering RAI treatment [30, 31]. Patients’ rationale for decisions about RAI appeared to be based on their understanding of the threat of their malignancy vs. the negative effects of the treatment, including uncertainty about benefits [32]. Other work among patients with papillary thyroid cancer (the most common histology) indicates patients differ in how they weigh both the urgency and pros and cons of thyroid cancer treatment options, and that reliable, trustworthy information is paramount for informed decision-making [33].

A robust literature supports the demonstrated efficacy of PtDAs for improving informed choice and decisional outcomes [34] among individuals making health treatment decisions [35]. The strongest effects are apparent for patients’ affective-cognitive outcomes [29]. When considering treatment options, patients with thyroid cancer benefit from a clear presentation of information about risks and benefits [17, 18]. Patients who are more involved in their decision report less regret over their treatment choice for thyroid cancer [19]. However, to date, we are unaware of any research that has focused on the development of a PtDA for patients with intermediate risk thyroid cancer.

Understanding patients’ perspectives about the RAI treatment decision-making process among patients with intermediate risk thyroid cancer may improve prospective treatment decision outcomes. The objectives of the current study were to elicit patients’ views of their decisional needs about RAI treatment decision-making to inform the design of a web-based PtDA for intermediate risk thyroid cancer patients.

## Materials and Methods

### Study Design

This qualitative study included patient focus groups, and data were collected between October 2021 and April 2022.

### Participant Selection and Setting

The Georgetown-MedStar Oncology Institutional Review Board (IRB #00004050) approved this study. Patients diagnosed with differentiated thyroid cancer (hereafter referred to as thyroid cancer) were recruited through referrals from endocrinologists and nuclear medicine specialists from three hospitals (two within a single academic medical center; one a community hospital) in the Washington, DC area. Patients were invited to participate in the study via email by study staff if they met the following inclusion criteria: (1) a diagnosis of differentiated thyroid cancer within the past 3 years, (2) were offered RAI treatment (regardless of ultimate RAI treatment uptake), (3) aged 18 years or older, and (4) had English language proficiency. A purposive sampling strategy was used to identify and invite participants to the study to the extent possible based on patient age (18 or older), sex (at least 40% male as thyroid cancer is diagnosed more often in women), RAI decision (RAI treatment or no RAI treatment), and referral site (across three different hospitals inclusive of four clinical practices (three endocrinology practices and one nuclear medicine practice). This sampling approach ensured the inclusion of individuals with direct experience of the RAI decision-making process, yielding relevant insights into the treatment-related informational needs. Eligible patients provided written informed consent with study staff via a HIPAA-compliant Zoom platform. The participants completed a brief online survey via REDCap to assess demographic and clinical variables before they participated in a virtual focus group discussion.

### Data Collection

The semi-structured interview guide (Electronic Supplementary Material 1) was developed by three authors with expertise in qualitative research (ALC), behavioral science (ALC, KDG), and endocrinology (JJ). The interview guide was informed by the Ottawa Decision Support Framework; this framework provides context for the development of DAs to guide patients through decisions with multiple treatment options, help address decisional needs, and improve decision quality in healthcare settings [36, 37]. The semi-structured interview included 11 open-ended questions with probes that explored patients’ (1) perceptions of the initial diagnosis of thyroid cancer, (2) knowledge and expectations throughout the RAI decision-making process, and (3) suggestions for a web-based PtDA for patients diagnosed with intermediate risk thyroid cancer.

On the basis of participant availability, the participants were divided into four focus groups with three to eight participants per group. In line with focus group sample size recommendations, a minimum of four focus groups is needed to reach data saturation or a benchmark of sufficient data collection to draw appropriate conclusions [38, 39]. The focus groups lasted 2 h and were held over a HIPAA-compliant videoconferencing platform. A clinical psychologist (ALC, female) moderated all four focus group discussions via a semi-structured interview guide for consistency across groups. A notetaker (either GP, research coordinator, male, or KLG, research coordinator, female) trained in qualitative research took field notes during focus groups. Focus group discussions were audio-recorded, transcribed verbatim, and de-identified. Focus group participants received a summary of the findings via email from the study team.

### Measures

#### Demographics and Clinical Variables

The participants reported their age, ethnicity, race, sex, marital status, education level, employment status, health insurance status, time since thyroid cancer diagnosis, RAI treatment decision (and, if applicable, time since RAI treatment), and type and stage of thyroid cancer.

#### Data Analysis

We analyzed the focus group data via conventional content analysis [40]. Two coders (ALC and KLG) with experience in qualitative research methods independently reviewed focus group transcripts and collaboratively developed a codebook via ATLAS.ti version 23.1.0 qualitative software. The two coders coded text that represented initial patterns or concepts and then synthesized the initial codes by consolidating the most salient codes into meaningful broad domains. The coders developed an initial codebook with detailed descriptions of the domains and refined the definitions through discussion until consensus was reached. The coders used an immersive organizational style in which the text was broken apart, individually reflected upon, and interpreted through domain and subtheme definitions. Intercoder consensus was reached through discussion [39]. Any overlapping subthemes were collapsed into fewer and more comprehensive categories [38]. The larger study team (JJ and KDG) reviewed and discussed the final interpretation of key domains and additional areas of consideration. The coders also discussed data interpretation and evaluated possible biases while investigating how well the synthesized data mapped onto existing empirical data or the team members’ clinical experiences. Guided by Krippendorff’s alpha coefficient, where *α* ≥ 0.80 is considered adequate [41], the intercoder reliability of *α* = 0.89 for the focus group data indicated adequate reliability. Using SPSS v 27, descriptive statistics were analyzed for sociodemographic and clinical variable data to characterize the sample.

## Results

### Participant Characteristics

**Fig. 1:**
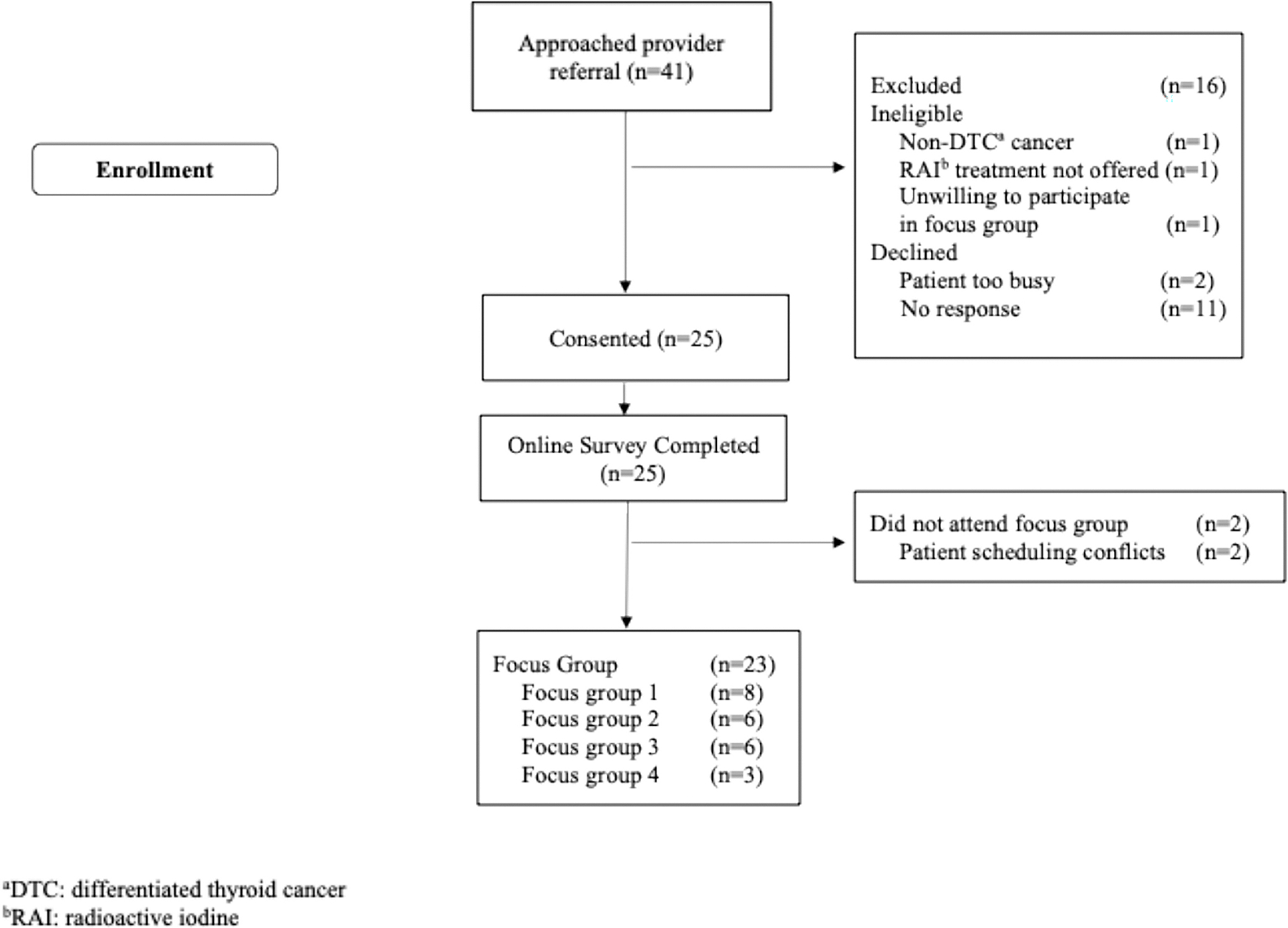
Qualitative focus group study flow chart

The study staff approached 41 patients for screening; 16 patients did not enroll (*n* = 2 ineligible, *n* = 11 lost to follow-up, and *n* = 3 declined, Fig. 1), and 25 patients with thyroid cancer consented and completed the online survey. Twenty-three patients participated in the focus groups. Two participants could not attend the virtual focus groups because of scheduling conflicts. The participants’ mean age was 39.1 years (SD = 13.5). Most of the participants identified as female (*n* = 19, 83%), non-Hispanic (*n* = 21, 91.3%), employed full-time (*n* = 20, 87%), and had a college education (*n* = 21, 91.3%). Participants self-identified their race as 21.7% Asian, 4.3% Black or African American, 13% as more than one race, and 60.9% as White. The average time since diagnosis was 1.7 years (SD = 0.94). Among participants (*n* = 19) who decided to undergo RAI treatment, the average time since treatment was 1.2 years (SD = 0.88). Most participants had a papillary thyroid cancer diagnosis (*n* = 19, 82.6%), and more than half of the participants did not know their cancer stage (52.2%). Table 1 shows patient-reported sociodemographic and clinical characteristics.

### Qualitative Content Analysis Results

Three broad domains emerged from qualitative analysis that represent the decisional needs of participants, which can inform a targeted web-based PtDA that is guided by the Ottawa Decision Support Framework: (1) the range of patient involvement in the treatment decision-making process, (2) personal value-based decisions, and (3) decision aid content recommendations on the basis of patients’ knowledge gaps. The domains and subthemes are described below, and exemplary quotes are provided in Tables 2, 3, and 4.

### Range of Patient Involvement in the Treatment Decision-Making Process

The participants described a range of personal involvement in their RAI treatment decisions (see Table 2). In particular, participants endorsed either shared decision-making with their provider or provider-delegated decisions (without patient involvement) as the most common level of involvement. Participants who endorsed shared decision-making indicated that their decision was based on their provider’s recommendations and their individual circumstances. Participants described shared decision-making as a positive experience and appreciated their provider’s guidance throughout the process. In contrast, the participants who characterized their RAI treatment decision as provider-delegated expressed the opinion that they were not given sufficient information about treatment options and felt pressured to follow the recommended treatment. A subset of these patients indicated that they were unaware that not receiving RAI treatment was a treatment option.

### Personal Value-Based Decisions

Participants shared how their personal values related to the physical, emotional, and day-to-day effects of treatment, and how the positive and negative treatment factors that mattered the most to patients influenced their decisions about RAI (see Table 3). The participants described their decision-making process as weighing the individual pros and cons of treatment options (to have RAI or not to have RAI). The most important factor among participants who decided to undergo RAI treatment was the prognostic benefit of the RAI. These participants emphasized that they valued specific anticipated outcomes of RAI treatment, such as the prevention of additional surgeries, the reduction in risk of disease-related recurrence, and the desire for prolonged survival, which ultimately led them to decide to undergo RAI treatment.

Conversely, many participants identified the risk of secondary cancer as a disadvantage of RAI treatment. Other patient-perceived drawbacks of RAI treatment include the risk of side effects, the potential need for multiple RAI treatments, and concerns about the impact of RAI on sexual function and fertility.

### Decision-Aid Content Recommendations from Patient Knowledge Gaps

**Fig. 2:**
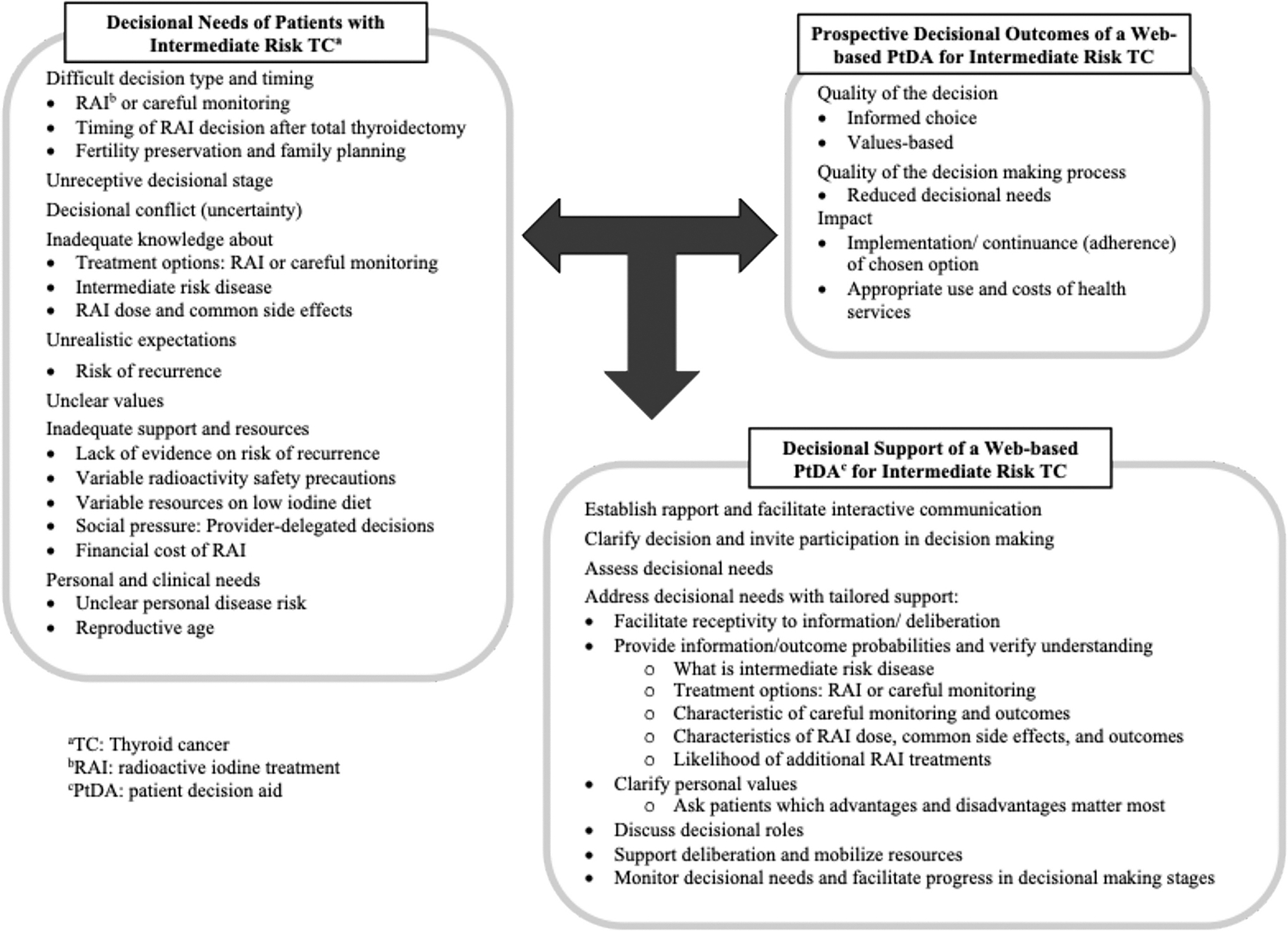
Patient recommendations for a web-based PtDA informed by the Ottawa Decision Support Framework[37]

The participants reported unmet decisional needs due to a lack of knowledge, resources, and unrealistic expectations about RAI treatment throughout the decision-making process. Participants provided recommendations for a prospective PtDA informed by their decisional needs (Fig. 2). Five subthemes emerged within this domain related to unmet decisional needs: (1) RAI dose and common side effects, (2) risk stratification, (3) radioactivity safety precautions, (4) the low-iodine diet, and (5) financial cost (see Table 4).

#### RAI Dose and Common Side Effects

Participants received insufficient information about the RAI dose–response relationship during the decision-making process. These participants wanted to know the magnitude of the RAI treatment dose, whether the dose influenced the type and severity of side effects, and whether the dose impacted the need for subsequent RAI treatment. Participants who received RAI experienced RAI-specific side effects, including jaw and salivary gland swelling and sinus problems, such as stuffy nose or nosebleeds, 6 months to 1 year after RAI treatment. Several participants expressed concerns about whether the side effects would continue long-term and uncertainty about whether thyroid surgery, post-surgical hypothyroidism, or RAI treatment contributed to these side effects.

Most focus group participants suggested that a web-based PtDA should include a list of common side effects, the duration of side effects, typical RAI dosing level ranges (e.g., high, medium, low), and side effects associated with doses to help patients feel more informed about their treatment decisions. Other participants requested information on the frequency of a second RAI treatment and a description of the tests or appointments needed before RAI treatment (e.g., pulmonary function tests or fertility preservation). The participants believed that this information would be important for patients with intermediate risk thyroid cancer to understand the risks and benefits of RAI treatment.

#### Risk Stratification

Several participants described the timeframe between thyroid surgery and the decision to consider RAI treatment as “quick” (Focus Group 2, PID 1), with little time for deliberation. They suggested that intermediate risk patients would benefit from a clear discussion with their healthcare team about the time sensitivity of the RAI treatment decision. The participants shared challenges understanding complex medical information about their risk level and confusion about the specific areas in the body targeted and/or affected by RAI treatment. Participants suggested that a PtDA should include visual information about the anatomical structure of the thyroid to help patients understand intermediate risk thyroid cancer and guide treatment decisions. Other participants recommended that a PtDA include a timeline for RAI treatment, such as the period after RAI administration to indicate when the medical team will know whether RAI targeted the intended areas of clinical concern. In addition, patients were interested in having access to information about how long they needed to be monitored (on average) before they knew they were “cancer free” (Focus Group 1, PID 7).

#### Radioactivity Safety Precautions

The participants commented on inadequate knowledge and expectations about radioactivity safety precautions following RAI treatment. They reported uncertainty about the post-RAI isolation period and the emotional and logistical implications of making treatment decisions. Multiple participants expressed loneliness during the isolation period and increased worry about exposing their loved ones to radiation as emotional implications of their treatment decision. The participants described the logistical implications of the treatment decision such as the use of plastic wrap and disposable items to prevent contamination, isolation at home without family or pets, regular washing of bedsheets, consistently cleaning the shower or bathtub after use, having a designated bathroom, abstaining from travel, and avoiding in-person interactions with vulnerable groups such as pregnant women.

Most participants stated that clear guidelines and explanations about safety precaution requirements, isolation duration, and risks would help patients with intermediate risk cancer consider whether they have the support and resources needed. The participants also recommended including timeframes when it is safe to return to work and engage in physical intimacy with a partner to better inform patients with intermediate risk cancer about RAI treatment expectations.

#### Low-Iodine Diet

The low-iodine diet was characterized as a short-term treatment burden among participants. The participants reported limited preparation for the low-iodine diet before treatment and were “surprised” (Focus Group 1, PID 8) by the lifestyle changes and resources required for RAI treatment preparation. Participants reported difficulties finding low-iodine recipes for preexisting dietary restrictions and inconsistent information from online resources on low-iodine safe foods. The participants suggested including a “*resources page*” (Focus Group 3, PID 4) in the decision aid with access to the low-iodine cookbook available through ThyCa: Thyroid Cancer Survivors’ Association, Inc. (ThyCa) to provide patients with adequate resources to make and implement their treatment decisions.

#### Financial Cost

The participants expressed mixed feedback on the extent to which the financial cost of RAI treatment informed their decision. Several participants reported that cost was not a significant factor in their treatment decision-making process and attributed their health insurance as a protective factor. In contrast, other participants identified cost as an important factor in decision-making. These participants reflected on the unanticipated costs of the isolation period immediately following RAI, such as separate lodging for family and pets, loss of wages, and the financial impact of taking time off from school. They emphasized that upfront knowledge of the anticipated additional costs and awareness of financial resources to help navigate healthcare expenses would be helpful to include in a PtDA for intermediate-risk thyroid cancer patients.

## Discussion

This qualitative study explored decisional needs from the perspective of patients with thyroid cancer offered RAI treatment to inform the development of a targeted web-based PtDA for intermediate-risk disease. Our findings suggest that insufficient knowledge and unrealistic expectations about RAI treatment outcomes contribute to decision-making difficulty. A PtDA, as an adjunct to the education and counseling provided by a physician, could help address these decisional needs by providing targeted decisional support to improve the quality of the decision-making process.

Our findings revealed a need for additional decision support, emphasizing the importance of personal values and the impact of insufficient knowledge and expectations on patients’ choices. Patients identified thyroid cancer risk categories, RAI dose, side effects, a low-iodine diet, and radiation safety precaution requirements as factors that impacted the quality of the decision. Patients expressed a preference for detailed descriptions addressing medical information and expectations about the physical, emotional, and social effects of treatment options. The participants prioritized the importance of their personal values in assessing treatment options, citing decreased mortality and the risk of RAI side effects as the most important advantages and disadvantages. The results suggest that a web-based PtDA presenting clear and balanced advantages and disadvantages of treatment options—RAI versus careful monitoring without RAI—would support informed and value-congruent patient decisions.

Another important finding was that patients reported limitations in timely access to relevant quality information about features of RAI treatment such as the low iodine diet and radioactivity period. These findings are consistent with those of previous studies, which indicate that patients with thyroid cancer often feel inadequately informed about aspects of treatment decision-making, including disease prognosis, potential side effects, and reproductive implications [22, 31, 42]. Notably, our study introduces a novel contribution by highlighting patients’ recommended awareness of logistical expectations associated with the RAI isolation period and the low-iodine diet. Knowledge gaps can negatively influence patients’ ability to make and implement treatment decisions, leading to greater dissatisfaction with treatment and poorer patient-reported quality of life outcomes [42]. DAs are well documented in the literature for their role in improving patient knowledge of treatment options and accurate perceptions of outcome probabilities, leading to more informed decision-making [22]. Our findings reinforce the importance of timely access to integrated risk information, personal values, and clear summaries of treatment options into the PtDA framework and expand on prior research by highlighting the importance of addressing logistical considerations of RAI treatment, including isolation and low-iodine diet requirements within PtDAs.

Shared decision-making is especially relevant for intermediate risk thyroid cancer patients, given the preference-sensitive recommendations for RAI treatment [15, 36]. However, patients with intermediate risk disease currently have limited supplemental resources to support this decision-making process. Our findings highlight patient preferences toward a targeted PtDA that aligns with the decisional support needs and personal values of thyroid cancer patients.

This study contributes to existing research by providing qualitative insights into contextual quantitative results on RAI-shared decision-making. A population-based survey revealed that more than half of patients did not feel they had a choice about RAI treatment [5], highlighting the medical complexities of RAI treatment [43, 44] and patients’ unmet decisional needs during and after medical encounters [3, 5, 45]. PtDAs can provide decisional support by offering structured information to facilitate patients’ understanding of medical information [36]. They can also be used to enable patients and clinicians to make informed quality decisions on the basis of patients’ values, thus balancing the benefits and risks of RAI treatment. PtDAs can be valuable tools for patients and providers to address uncertainty, bolster realistic expectations, and provide targeted support.

A limitation of the study is that most participants received RAI treatment, and only one-fifth chose careful monitoring with no RAI treatment. The results may not be fully generalizable to patients who choose careful monitoring with no RAI treatment nor fully capture these patient perspectives in the decision-making process. Likewise, given our purposeful sampling approach and the guided focus group questions, the results may not have captured all of the reasons participants may have opted to have, or not have, RAI treatment. Given that the average time since treatment was 1.2 years, participants’ accounts of their decision-making needs may have been potentially influenced by recall bias. We did not elicit information about the dose of RAI given among those who received this therapy. In addition, approximately half of the participants did not know their stage of cancer or risk category, which indicates that patients may benefit from medical information about their specific diagnosis, along with treatment options on the basis of their thyroid cancer risk level. Additionally, our sample was relatively homogeneous in terms of patient self-reported race and ethnicity, and all participants had health insurance. Future work should focus on understanding decision-making processes from patients’ perspectives from different racial, ethnic, and socioeconomic backgrounds to characterize their decisional needs. In addition, attention to patients’ health literacy and ability to understand risk information will be important for the development of PtDAs to support RAI treatment decisions [46]. Notwithstanding these limitations, the results provide insights from patient perspectives to inform the development of a web-based PtDA among patients with intermediate risk thyroid cancer.

Given the shifting landscape in which RAI treatment is definitively recommended for high-risk patients with thyroid cancer and not usually recommended for low-risk patients, the idea of patient choice is a new and challenging concept for patients with intermediate risk thyroid cancer. High-quality patient-facing information on the medical risks and benefits of RAI treatment is needed to support patient-centered RAI treatment decisions. The development of an accessible tool, such as a targeted web-based PtDA, may offer adjunct support in the decision-making process for patients with intermediate risk disease.

## Supplementary Material

Supplementary Material

**Supplementary Information** The online version contains supplementary material available at https://doi.org/10.1007/s12529-025-10408-4.

## Figures and Tables

**Table 1:** Patient-reported sociodemographic and clinical characteristics (*N* = 23)

Characteristics	*N* (%) or M (SD) *N* = 23

Age in years M, (SD; range)	39.1 (13.5; 22.4–71.3)
Ethnicity	
Hispanic or Latino	2 (8.7)
Non-Hispanic or Latino	21 (91.3)
Sex	
Male	4 (17.4)
Female	19 (83)
Married/partnered	9 (39.1)
Race	
Black or African American	1 (4.3)
White	14 (60.9)
Asian	5 (21.7)
More than one race	3 (13)
Education	
Some college	2 (8.7)
≥College	21 (91.3)
Employment Status	
Full-time employed	20 (87)
Part-time employed	1 (4.3)
Not employed	1 (4.3)
Retired	1 (4.3)
Health insurance, yes	23 (100)
Time since diagnosis in years, M (SD; range)	1.7 (0.94; 0.1–3.0)
Time since RAI^a^ treatment in years,^b^ M (SD; range)	1.2 (0.88; 0.1–3.0)
Diagnosis type	Frequency
Papillary	19 (82.6)
Follicular	1 (4.3)
Other	2 (8.7)
Don’t know	1 (4.3)
Stage	Frequency
I	8 (34.8)
II	2 (8.7)
III	1 (4.3)
Don’t know	12 (52.2)

a RAI: radioactive iodine

b Four participants did not receive RAI treatment

**Table 2:** Exemplar quotes related to patient involvement in the treatment decision making process theme

Domain 1: Range of patient involvement in the treatment decision-making process		RAI uptake Yes or no
Description	Exemplar quotes	

Shared-decision [relating to making a treatment decision with their provider, back and forth conversation with provider about the decision, choice of the options is known to the patient]	*What led me to do it [RAI treatment] is because I trusted my doctor* Focus Group 1, PID 3	Yes
	*I had a choice…my doctor said it’s up to you basically you know these are the pros and cons, and it will, you know, help kill any cancer cells that may be, you know, still in there. After the surgery, about the large cancer. And it's spread in three or four lymph nodes that they take out.* Focus Group 1, PID 7	Yes
	*After the surgery, [thyroid surgeon’s name] sat me down and said that it was really clean. She took out everything. She didn’t think that I required RAI and it’s of course my decision.* Focus Group 4, PID 3	No
Provider designated decision [reference to not knowing there was a decision to not have RAI, provider telling patient the decision to make]	*I did not know that there was a choice. They were like, “You have to do this. ” I didn’t know that you could not do it*. Focus Group 1, PID 1	Yes
	*It wasn’t so much about whether should I make the decision, it was more like the decision was made. It was like what’s going to happen, where should I have it.* Focus Group 2, PID 4	Yes
	*They just told me, you should get RAI. And so, I did.* Focus Group 4, PID 2	Yes

**Table 3:** Exemplar quotes related to personal values-based decisions domain

Domain 2: Personal value-based decisions		Had RAI treatment Yes or No
Description	Exemplar quotes	

Advantages of RAI treatment [perceptions of benefits of RAI treatment including a pathway to remission, perception of lower likelihood of recurrence, peace of mind]	*[There] wasn’t a lot of options. I wanted to extend a little bit of time that I have left and do as much as I can*. Focus Group 1, PID 4	Yes
	*I have been under surveillance and monitoring, and God forbid there’s a recurrence at this point. If I needed to, I would be able to do RAI. I would not hesitate if that were the recommendation if I needed it because there was a recurrence, I would absolutely do it.* Focus Group 2, PID 1	No
Disadvantages of RAI treatment [practical life considerations and long-term concerns about health risks including risk of secondary cancers, acute and long-term side effects, disruptions to daily life]	*I’m in the middle of fertility therapy, so [I] was waiting. I asked [endocrinologist’s name] many questions… Should I go without RAI, or what will happen to my fertility? I’m under a time constraint, and I’m supposed to have my egg retrieval. Should I start with it? Which should I do first? Surgery, retrieval, and then [RAI]?* Focus group 1, PID 6	No
	*I asked my doctor a lot of questions because when he said that there is a risk of developing salivary gland cancers down the road, even though it is a very small risk, that scared me. When I was looking at the prognosis, the chances of survival from salivary gland cancer* versus *thyroid cancer are much more. I had a few weeks where I was not sure if I should go with this [treatment] but eventually ended up taking my doctor’s advice.* Focus Group 1, PID 8	Yes
	*I do not want to risk other cancers and other side effects just to find that out. To me, it was an obvious weighing on the scale, but to her [nuclear medicine provider], I do not know that it was interesting.* Focus group 2, PID 2	Yes

**Table 4:** Exemplar quotes related to patient knowledge gaps domain

Domain 3: Decision aid content recommendations from patient knowledge gaps		Had RAI treatment Yes or No
Subtheme	Exemplar quotes	

RAI dose and common side effects [relationship between RAI dose and side effects, common side effects occurring after RAI treatment, RAI side effects impact on lifestyle]	*The side effects depend on the amount of dose that you receive. Having [a] range along with the side effects that you may get with that dose, so at least you are prepared.* Focus Group 1, PID 7	Yes
	*I had a clogged gland, and the side of my face swelled up. They do not talk about those long-term things in the grand scheme. I’m alive… I did not know this was going to happen. I think that long-term care, as you’re going through it, should be explained more.* Focus Group 2, PID 4	Yes
	*I want to taste an apple. That’s not the biggest thing, but for some people, that might be critical. You have the chance to lose your sense of taste.* Focus group 3, PID 1	Yes
Risk stratification [patient understanding of low, intermediate, high risk for thyroid cancer; areas of the body targeted from RAI based on risk type]	*There’s one piece of basic science that truly confused the hell out of me, and I truly wish someone had explained this. Not understanding the science truly threw me for a loop because then it was like, “If the RAI worked, then how could I still have cancer?”* Focus Group 2, PID 2	Yes
	*I feel like sometimes the way doctors talk is like they’re talking to other scientists. In addition, you’re like, “Wait, what does that mean?”* Focus Group 3, PID 4	No
	*Who makes that decision if you are low risk or intermediate risk?* Focus group 4, PID 3	No
Low iodine diet [reference to patient preparation, credible resources and recipes, patientprovider communication about preparation]	*The information online was contradictory. For example, egg whites. On some websites, they were saying, “It is low iodine; you can have it. No problem.” Other websites that I [went] on that were not affiliated with [institution name], for example, would say, “Even egg whites have iodine, so you should not incorporate that into your diet.”* Focus Group 1, PID 8	Yes
	*The diet can also be a challenge having to plan out. There is so much to be so aware of [and] everything that you’re eating*. Focus Group 2, PID 6	Yes
	*My salt levels were so low, I got very sick… I’d do anything for a couple weeks, but it truly made me sick. In addition, what I recommend to someone… I think that is a personal decision I think people need to listen to their bodies and to their doctor*. Focus group 3, PID 2	Yes
	*There’s a lot of planning involved. I cannot travel for a certain amount of time before-hand because of the diet, and I cannot travel for a certain amount of time after.* Focus Group 3, PID 6	Yes
Radioactivity safety precautions [logistics needed to ensure radioactivity safety during isolation period]	*They tell you if you have kids that… you should not be around kids. However, at the time, I had a newborn niece, and I was scared. I was like, I’m going to double that time. In the end, I did not see her for two months. Those emotional things are hard. It was a choice that I made, but I think [it is] so unknown [that] you do not want to hurt anybody else*. Focus Group 2, PID 4	Yes
	*When eating, use paper plates, washing down the bathroom, and stuff like that. I think that would be very, very helpful for the web page*. Focus Group 3, PID 2	Yes
	*You need very specific circumstances for it to be workable [isolation period]. You need space to be alone, your own bathroom or access to your own bathroom. No pets, kids, or pregnant women*. Focus Group 3, PID 5	Yes

Financial cost [financial impact and considerations of RAI treatment]	*For me, I was like, I don’t care what it costs; I want to [get rid of] this cancer. However, then, when I made the decision, you do have to navigate the cost*. Focus Group 2, PID 4	Yes
	*Cost [had] a little bit of a factor in the sense of I had it [RAI] last year. I was very young, under my family’s insurance, and my family took care of me during COVID, so [school was] completely online. I was still able to do my school [work]. I was in the middle of college, and I did not have to put my life on hold to get this treatment*. Focus Group 3, PID 5	Yes
	*I think you have to talk about [the] cost because it’s a big part of it, but you don’t want costs to be stopping people from doing it [RAI treatment] who otherwise would. If there’s anything that can help people with that, have that information on there*. Focus Group 4, PID 2	Yes

*RAI* radioactive iodine

## Data Availability

No datasets were generated or analysed during the current study.
