# Supplementary material for: Patient Perspectives Toward a Decision Aid for Radioactive Iodine Treatment for Intermediate Risk Thyroid Cancer
Source: Int J Behav Med. Author manuscript; Available in PMC 2026 Apr 14. (PMC13077738; doi:10.1007/s12529-025-10408-4)
Supplement: Supplementary Material [file NIHMS2153144-supplement-Supplementary_Material.docx]

**Focus Group Moderator’s Guide – RAI Decision Aid Focus Group Discussions**

**Instructions**: This guide is semi-structured—it includes questions and prompts that we will ask in the focus group discussions, but additional questions and prompts will be used depending on the topics, ideas, and themes that arise in the discussions. The questions and prompts shown below are representative of the topics to be covered and elaborated upon in the groups.

Note: These groups will be conducted via video-conference

**Procedures**:

1. Project staff will greet individuals as they enter the video conference room and check off their name on the attendance sheet.
2. When it is time to begin, the moderator will offer another warm welcome and will provide an explanation of the purpose of the focus group meeting. The moderator and any other project staff in attendance will introduce themselves.

**Section 1: Start Group (10 minutes)**

*Thank you for being here. We want to welcome you today and share our appreciation for your time and willingness to participate. My name is* **_____** *and today I will be leading our focus group discussion. A focus group is a meeting where questions are asked to a group of people about a specific topic. Researchers use focus groups to learn about a topic from people who have first-hand experience with it. The purpose of this focus group is for us to listen to your thoughts and feedback about your thoughts and decisions related to treatment for thyroid cancer. We are specifically interested in your thoughts and decisions about having radioactive iodine therapy, which we will abbreviate as RAI during this discussion.*

*You should feel free to make any sort of comments – positive or negative – about what we are talking about today. There are no right or wrong answers. We truly appreciate your help. We are interested in finding ways to help support patients who will be making decisions about RAI and all experiences are valuable as we learn more about how these decisions are made.*

*1.* The moderator will briefly remind participants of key sections of the consent form and answer questions.

*We emailed you a consent form to read and sign and received it back from all of you, along with a brief survey. Right now I’d like to take a moment to review the main points of the consent form and answer any questions you have. Remember that your participation is completely voluntary and you can decide you want to stop participating at any time.*

Important points to note:

- Protection of privacy
  - Be sure to note that today’s discussion will be recorded. If you do not feel comfortable with this, please send a chat message to [STAFF MEMBER].
  - You can choose to skip any questions you do not wish to answer; you can simply say “pass” if we ask for your input you and you do not wish to answer.
- Who to contact with questions after the group is over.

*Are there any questions? Are you all still willing to continue? And to be recorded?*

2. The moderator will review the ground rules.

*We want everyone to have the chance to share their opinions or experiences. We hope that this will be a very open discussion. There are just a few ground rules we want to go over that will help everything go more smoothly:*

- *Talk one at a time.*
- *Be respectful of others. You don’t have to agree with what’s said.*
- *Keep today’s discussions private. What is said in this videoconference should stay here.*
- *If you do not want to answer one or more questions, that’s okay. You don’t have to and as we shared earlier, you can simply say “pass.”*
- *Please keep your camera on if you feel comfortable, and mute when you are not talking to reduce background noise.*
- *Feel free to use the zoom ‘reaction’ emojis if you wish (located under the reactions button at the bottom of the page).*
- *Please do not take any screen shots to protect people’s privacy.*
- *We may call on people to make sure we hear different opinions and perspectives.*
- *Does anyone have any questions? Great. Now I’ll start recording.*

**(START RECORDING)**

**Section 1: Icebreaker / Opening Discussion (20 minutes)**

1. Ask them to introduce themselves, first name only, where they are located and one thing you like about summer.

2. *Think back to when you were diagnosed with thyroid cancer. Looking back, was there anything that took you by surprise or that you wished you had known about ahead of time?*

**Section 2: RAI Decision Making (35 minutes)**

1. We are interested in your experiences with making decisions about RAI.

*1a: What comes to mind when you think about what influenced your decision to have, or not have, RAI?*

*1b: What information would you have liked to have before RAI treatment?*

*1c. Did you seek out information on your own about the pros and cons of RAI?*

*If so, where did you seek information from? (probes: WebMD, ThyCa, general searches, etc.)?*

*1d. Who on your clinical team, if anyone, talked with you about RAI?*

*1e. Who else did you talk with about this decision? Family? Online or in person support groups?*

2. We also would like to hear what information you think other patients should know when making a decision about RAI?

*2a.What are the factors you consider most important about making a decision about RAI?*

- *Medical / therapeutic benefits?*
- *Logistics of having RAI?*
- *Side effects?*
- *Costs*
- *Short and long-term effects?*

*2b. What do you see as the ‘pros’ of having RAI?*

*2c. What do you see as the ‘cons’ of having RAI?*

*2d. Would you recommend RAI to someone else? Or other treatment approaches?*

1. We are interested in developing a web-based decision aid to help support patients diagnosed with **intermediate** **risk thyroid cancer** who are making decisions about whether to have RAI or not. By intermediate risk, we mean a group of patients for whom RAI may not have clear benefits one way or the other.

*3a. We are thinking of including information in the decision about different topics. We will go through this list of topics and would like to hear your thoughts about each one. [Pause after each topic and ask “is this useful to include on a website to help people make decisions about having RAI or not?]*

- - *the factors that make someone a good candidate for RAI*
  - *the pros and cons of having RAI,*
  - *the types of symptoms some patients experience over time,*
  - *how to consider the decision of RAI in relationship to your medical treatment goals*
  - *concerns about side effects (mouth, salivary glands, eyes, nose)*
  - *concerns about financial costs (managing side effects, dental problems)*
  - *concerns about social effects (dry mouth, talking, not tasting certain foods)*
  - *concerns about effects on fertility and family (delaying plans for conception, keeping family safe)*
  - *concerns of causing another cancer*

**Section: Symptom Burden and Support (10 min, if there is time)**

- 1. At any point in your RAI treatment, did you feel you needed more information on how to manage treatment related symptoms?

1a. If yes, what information would have been helpful at the time?

- 1. At any point in your RAI treatment, did you feel you needed more support on how to manage stress?

2a. If yes, what type of support would be most helpful?

1. From whom or in what format?
   1. What symptoms were the most challenging to manage after RAI?

3a. Probe: Short-term symptoms (e.g., nausea and vomiting, dry mouth, taste changes, neck tenderness and swelling, swelling and tenderness of the salivary glands)

3b. Probe: Long-term symptoms (e.g., tiredness, inflammation of salivary glands, dry or watery eyes)

- 1. Describe the ways you managed these symptoms.
  2. Are there symptoms that you are still struggling with that have yet to be resolved?

**Section: Closing (5 minutes)**

*Based on our discussion today, what do you feel are two main points I should take back to our team?*

*Is there anything else you feel we did not cover that we need to know?*

*We would like to thank you for your time.  Your answers have greatly helped us.  If you have any questions about what we have done today, do not hesitate to call the phone number on the bottom of the consent form.  We will be happy to talk with you about the study. Also, if anyone would like a summary of results from the focus group, we would be happy to provide a summary. We will make sure to take out anyone’s name or other identifying information. It will be a few months before the summary results are ready, but we are happy to share with anyone who is interested. You can email study staff to indicate interest or to ask any follow up questions [post email address in the chat].*

**(STOP THE RECORDER)**

**[Discuss distribution of incentives and paperwork]**
